# The fading popularity of a local ecological calendar from Brunei Darussalam, Borneo

**DOI:** 10.1186/s13002-022-00525-9

**Published:** 2022-04-16

**Authors:** Nurzahidah Bakar, F. Merlin Franco

**Affiliations:** 1 Faculty of Arts and Social Sciences, Universiti Brunei Darussalam, Bandar Seri Begawan, Brunei; 2 Institute of Asian Studies, Universiti Brunei Darussalam, Bandar Seri Begawan, Brunei

**Keywords:** Phenological knowledge, Landscape management, Ecosystem services, Calendar keeper, Indigenous calendar

## Abstract

**Background:**

Local ecological calendars are ecocultural frameworks that link temporal and spatial scales, contributing to resilience and adaptive management of natural resources and landscapes. They also facilitate management, access and withdrawal of provisioning ecosystem services. In this article, we describe how the ecological calendar of the Kedayan people of Brunei Darussalam links skyscape and biodiversity with sociocultural aspirations to foster adaptive management of landscape, and provide an understanding of the transmission of calendric knowledge in the community.

**Methods:**

In 2018, we collaborated with sixteen purposively sampled knowledge keepers from the Kedayan community of Brunei Darussalam to document the Kedayan local ecological calendar, and develop a calendrical pictogram. Using a structured questionnaire, we then interviewed 107 randomly selected community members, to understand the contemporary relevance and popularity of the Kedayan calendar, and the transmission of calendric knowledge in the community.

**Results:**

Our findings reveal that very few respondents (*n* = 27, 25.3%) are aware of the existence of Kedayan ecological calendar; majority (*n* = 80, 74.7%) were not aware of its existence. There is no statistically significant correlation between consulting healers, knowledge on appropriate time requisite to consult healers, and awareness and self-professed knowledge of Kedayan calendar. Only 14 (13.1%) of the respondents reported to have received some form of calendric knowledge, while the majority (86.9%; *n* = 93) never received any calendric knowledge. Only a negligible 1.9% reported to have transmitted calendric knowledge to others indicating a breakdown in transmission of calendric knowledge.

**Conclusion:**

The calendric pictogram would help the community in revitalizing their calendar. However, the community will have to invest on enhancing transmission of calendric knowledge.

**Supplementary Information:**

The online version contains supplementary material available at 10.1186/s13002-022-00525-9.

## Introduction

In the 1960s, when *angin punay—*an alternatingly strong and calm wind brought the emerald doves to the landscape inhabited by the Kedayan community, Haji Ali, a farmer back then knew it was time to scout for the rising of Pleiades in the east. He woke up in the mornings and walked to his open field from where he could observe the star cluster, the rising of which signaled the time for preparing the field for paddy cultivation. Today, an elderly Haji Ali does not scout for Pleiades anymore even when *angin punay* has arrived, as modern weather forecasting system together with improvised cultivars have weaned the Kedayan farmers away from their local ecological calendar (LEC). For the majority Kedayan who have shifted away from their traditional occupation of subsistence farming [1, 2], the Gregorian and Islamic calendars suffice to plan their civil and religious activities, respectively, and the need for checking weather forecasts seldom arises.

Haji Ali is an elder of the Kedayan community who are indigenous to Brunei Darussalam and northern Borneo [3]. They are rarely referred to by their autonym Urang Darat, as the name Kedayan/Kadayan has become popular throughout Borneo [2]. The Kedayan language is closely related to the Brunei Malay language, with high lexical similarity. Consequently, many scholars treat both Kedayan and Brunei Malay as the same [4, 5]. The Kedayan are one of the few Islamic communities of Borneo who have retained some pre-Islamic indigenous cultural practices, including rituals and festivals; in the neighboring Malaysian Borneo, they have been relentlessly targeted by research studies of questionable ethical standards that accuse them of syncretism [6–8]. In the past, the Kedayan of Brunei practiced both shifting and permanent cultivation of rice, planting local cultivars best suited to the local ecological conditions. Sago cultivation, though not extensive as rice cultivation, was also practiced [2]. Unlike the politically powerful Barunay (Brunei Malay), the Kedayan did not fear the forests, but depended on it for provisioning ecosystem services such as medicinal plants, wild fruits, vegetables, construction materials and game. Meat, especially that of wild animals, was culturally important for the Kedayan as meals devoid of them were considered of lesser quality [2]. Associated with subsistence farming, foraging, hunting and fishing in the past [9, 10], the Kedayan were the major suppliers of food to the Brunei Malays. This relationship has changed as most Kedayan have shifted to formal employment opportunities offered by the oil- and gas-based economy [2, 11]. In the 1970s when Allen Richmond Maxwell Jr. [12] conducted his landmark study ‘Kadayan Ideas of Time,’ many Kedayan were actively practicing agriculture, making use of their local ecological calendar (LEC). They watched the opening and closing of *picula* flowers (*Luffa* sp.) to determine the time of the day and plan the day’s agricultural activities accordingly. They had also scouted for star clusters such as Pleiades to plan their agricultural activities. While many communities in the Malay archipelago used solar gnomons to track the sun’s movements, the practice has not been reported from the Kedayan [13].

Local ecological calendars (LECs) such as the Kedayan calendar are not mere instruments to determine time [14]. For local communities, they are ecocultural frameworks that link temporal and spatial scales, contributing to resilience and adaptive management of natural resources and landscapes [14–18]. As ecocultural frameworks, they also facilitate management, access and withdrawal of provisioning ecosystem services through fishing, foraging, hunting etc. [19], a role that is often overlooked. LECs utilize changes in the skyscape and landscape, including biological rhythms of flora and fauna as temporal markers which is then linked to the social and cultural rhythms of the community. Folklores in local languages transmit calendric knowledge [20], while local languages also possess lexemes with layers of meanings to denote the event-based time intervals [21, 22]. Thus, LECs also form important components of biocultural diversity [23]. In the twentieth century, codified calendars were studied mostly for their role as instruments of power [24], relationship with social organization [25], influence over cityscapes [26, 27], astronomical knowledge and agricultural productivity [28], calendrical devices [29], etc. The beginning of twenty-first century saw a renewed interest in calendars, but with a focus on LECs that are disappearing, perhaps at a faster pace than local languages, culture and biodiversity. Breakthrough research studies prove that these calendars are repositories of local knowledge that can foster natural resource and landscape management [15–17, 30], climate change adaptation/mitigation [31–35], hunting and foraging [36], etc. Community-led projects for documentation and revitalization of LECs can stem knowledge erosion, while also offering time-tested adaptive ecocultural frameworks for natural resources and landscape management, and climate change adaptation rooted in the local cultures [37, 38]. During January to June 2018, we collaborated with the Kedayan people of Brunei Darussalam to document their LEC with the goal of revitalizing it, and generate an understanding of the transmission of calendric knowledge in the community. The specific objectives were: (i) to understand the ecological importance of the calendar, (ii) to uncover the areas of intersection between folk medicine and LEC, and (iii) to investigate the transmission of calendric knowledge in the community. In this article that originates from the study, we provide an account of the Kedayan LEC, and highlight its ecological significance in linking skyscape and biodiversity with sociocultural aspirations to foster adaptive management of landscape. We then trace transmission of calendric knowledge in the community, to provide an understanding of the meager community transmission that has contributed to the fading popularity of the Kedayan LEC. The article is among the very few that investigate the transmission of folk calendric knowledge in the community [39]. Part of the study that deals with the intersection of Kedayan folk medicine and LECs is in press at the time of drafting this article [40].

## Materials and methods

We adopted a mixed method approach involving both qualitative and quantitative data [41]. We gathered qualitative data to document the Kedayan ecological calendar, and quantitative data to find out the popularity of the calendar and the transmission of calendrical knowledge.

### Documentation of Kedayan ecological calendar

**Fig. 1:**
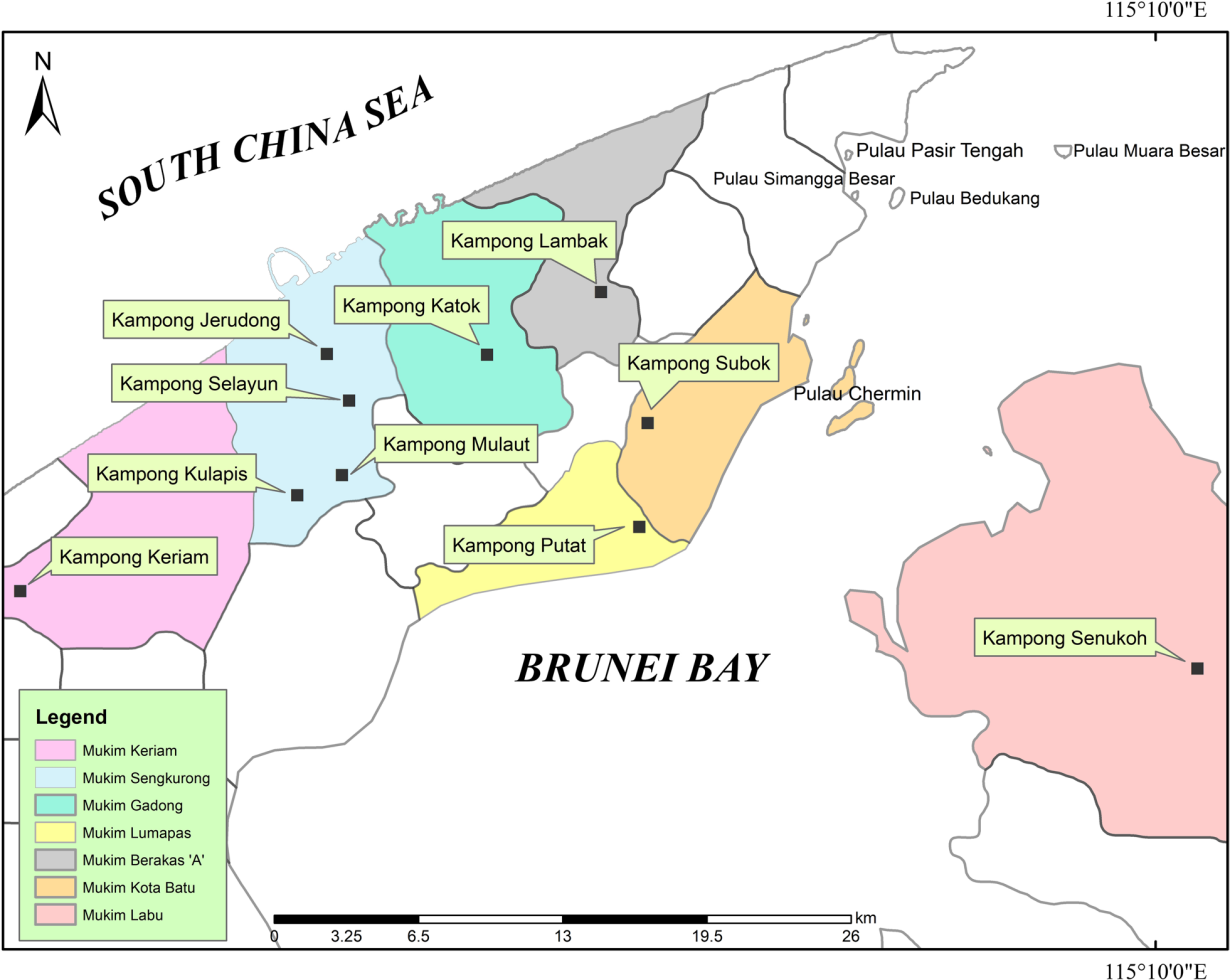
Map showing localities where interviews with knowledge keepers were conducted

We identified 16 knowledge keepers from the community within Brunei using purposive sampling (Fig. 1). Our knowledge keepers are mostly males (13/16), with ages ranging from 52 to 95, reputed for local knowledge related to Kedayan LEC and healing practices. Data were collected through in-depth interviews using a semi-structured questionnaire by the first author who is a member of the Kedayan community. Questions elicited information on months and seasons, astronomical knowledge, local seasonal indicators, application of the calendar, calendric knowledge related to agriculture, folklores and taboos. Results were compared to Maxwell’s study on Kedayan time keeping, and used to reconstruct the Kedayan ecological calendar [12].

### Interviews for determining the relevance and popularity of the Kedayan ecological calendar, and its transmission in the community

**Fig. 2:**
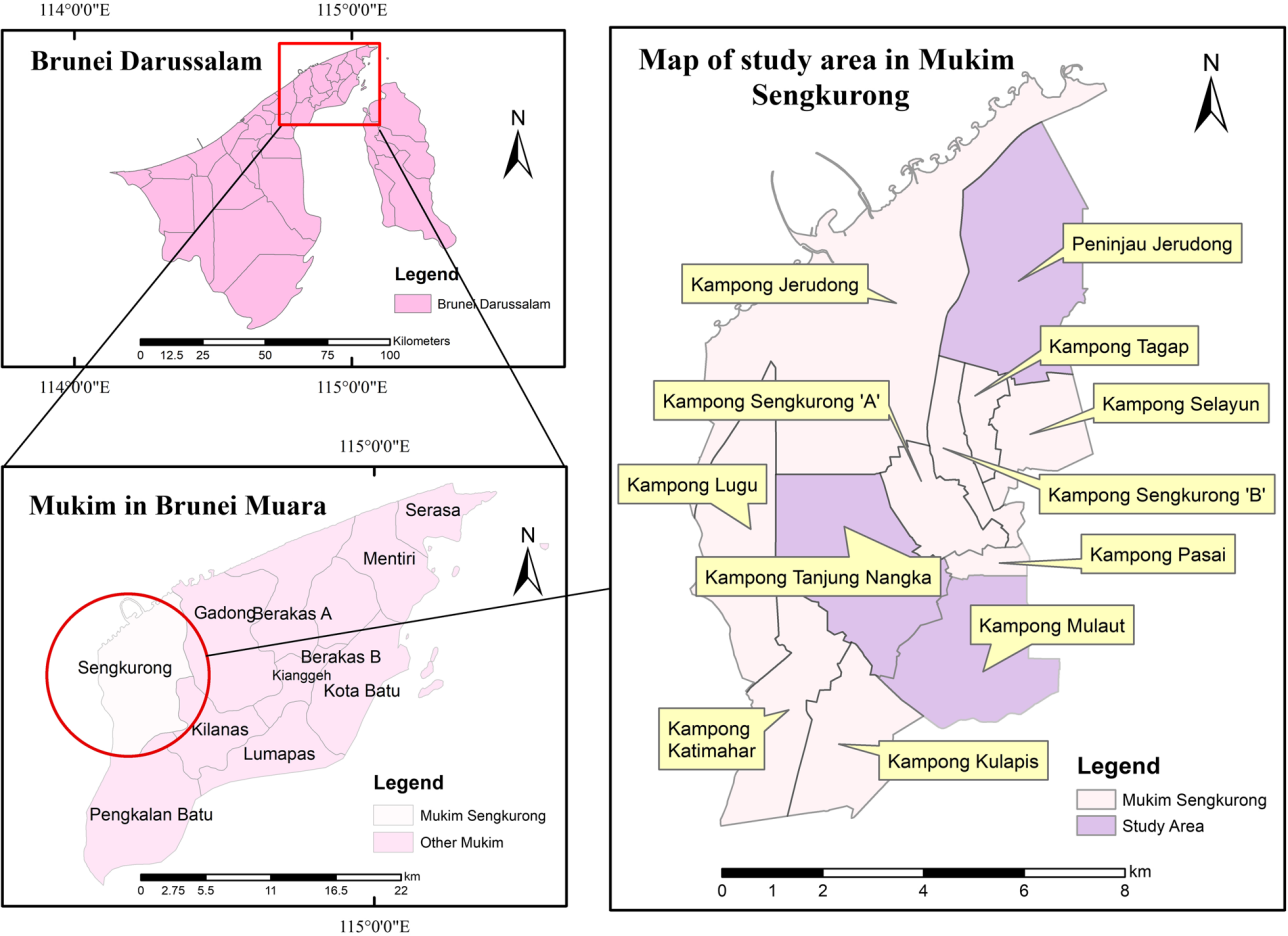
Map of Mukim Sengkurong showing localities where respondents were interviewed using structured questionnaire

We used structured questionnaire to find out the contemporary relevance of Kedayan ecological calendar (Additional file 1). This part of the study focused on Mukim Sengkurong (Sengkurong Sub-District) purposively selected due to the high population of Kedayan community. Within this Sub-District, the lead author distributed questionnaires to three selected villages, viz., Kampong Peninjau Jerudong, Kampong Mulaut and Kampong Tanjung Nangka (Fig. 2). Within the villages, we employed systematic random sampling to identify respondents, inviting every third household in the village to participate in the study. When the third house was empty or declined to participate, the lead researcher moved on to the sixth house, and so on. The questionnaire was organized into two sections. Section A elicited data on the personal particulars of the respondents (Age, gender, villages, educational and occupational background), while section B covered respondents’ knowledge on Kedayan calendar, flow of calendric information. A total of 107 respondents participated in this phase (Table 1).

**Table 1:** Demographic particulars of respondents who answered structured questionnaire on the relevance and popularity of the Kedayan ecological calendar, and its transmission in the community

Number of respondents	107 (males = 44; females = 63)
Age groups	
< 18	10
19–29	47
30–39	15
40–49	8
50–59	20
60–69	6
70 above	1
Villages (location)	
Peninjau Jerudong	33
Mulaut	32
Tanjung Nangka	42
Educational level	
Primary	2
Secondary	44
Diploma (tertiary)	23
Undergraduate	24
Postgraduate	10
Phd	1
Other (not available)	3
Occupation	
Government	36
Private	28
Business	0
Agriculture	0
Hunting	0
Fishing	7
Herbalist	0
Other (not available)	36

### Research ethics

We conducted risk assessment, and obtained ethical approval from University Research Ethics Committee of Universiti Brunei Darussalam (Ref. UBD/FASS/ETHICS/2018/FEB-01) prior to the study. The research also conforms to the code of ethics in ethnobiology [42]. We provided an information sheet with particulars of the study to the participants, and obtained their prior consent. To ensure anonymity of participants, we have only used pseudonyms in this article.

## Results

We reconstruct the Kedayan local ecological calendar in “Kedayan local ecological calendar” section using primary data from the qualitative interviews, and secondary data from literature [12]. In “Contemporary relevance and popularity of Kedayan ecological calendar” section, we present the results of structured interviews that provide an understanding of the popularity of the calendar in the contemporary Kedayan society. Transmission of calendric knowledge is presented in “Transmission of calendric knowledge” section.

### Kedayan local ecological calendar

**Fig. 3:**
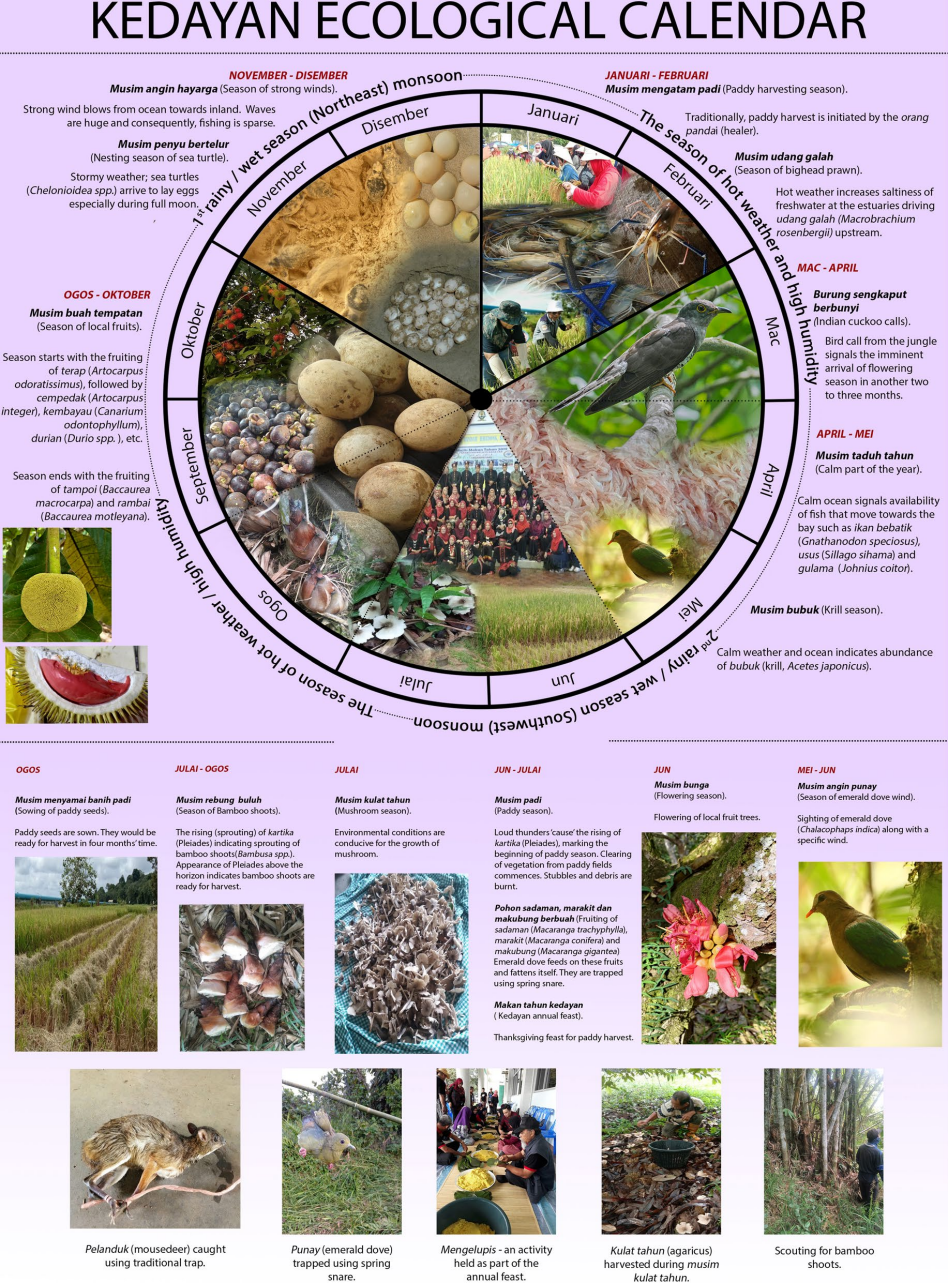
Pictogram of Kedayan dynamic local ecological calendar. *Photograph sources*: Joremy Tony (Emerald Dove), CC-BY-SA-4.0 Rejaul Karim (Indian Cuckoo), Nurzahidah Bakar (All others). *Note*: Names of months are in Kedayan/Bahasa Melayu

The Kedayan LEC of the past was a calendrical complex comprising indigenous solar, stellar and lunar calendars. The stellar calendar together with local seasonal indicators helped the community determine appropriate timing for landscape management activities, especially agriculture. The lunar calendar and local seasonal indicators guided fishing, hunting and foraging activities, and planning of sociocultural events such as festivals and rituals. *Makan tahun* (harvest/thanksgiving) festival is the only indigenous festival linked to the stellar calendar today. In the Kedayan LEC that survived in to the twentieth and twenty-first centuries, the lunar calendar was replaced by the Islamic calendar, and solar calendar by the Gregorian calendar. We could not document any folk songs, stories and riddles related to Kedayan ecological calendar despite our best efforts. Much of oral calendric knowledge that has survived today is passive, with the knowledge on star clusters and constellations limited to a few knowledge keepers. We could retrieve no knowledge related to the solar calendar, indicating that it has been completely lost long ago. The Kedayan LEC presented in Fig. 3 and Table 2 depicts the elaborate LEC embedded in the calendar and the sociocultural activities influenced by it. The Kedayan reckoned time and seasonal cycles by observing events in the skyscape (sun, stars and moon) and landscape (local seasonal indicators). Contingent on the fixed lunisolar cycles and the fluctuating local seasonal indicators, the Kedayan seasonal cycle is dynamic and different from the fixed seasons represented in the Gregorian calendar. In the Kedayan calendar, wet phases of the year alternate with hot weather of high humidity. Each phase is further divided into *musim* (seasons). Kedayan recognize sixteen *musim* in a year.

**Table 2:** Kedayan local ecological calendar and the ecological events that serve as local seasonal indicators

Gregorian months	Musim (season)	Seasonal indicator		Events (landscape management and withdrawal of provisioning ecosystems services)
		Celestial indicator	Local seasonal indicator	
January–February	*Musim mengatam padi* (Paddy harvest season)	–	–	Season when the paddy is mature and ready for harvest (around January to February). For the Kedayan community, the harvesting of paddy was usually initiated by the *orang pandai* (healer), followed by other members of Kedayan community. The Kedayan associate harvesting season with happy mood
	*Musim udang galah* (season of bighead prawn)	–	Increasing saltiness of freshwater	Season of hot weather when freshwater turns salty, driving upstream migration of bighead prawns (*Macrobrachium rosenbergii*). It is the optimal time to harvest bighead prawn
March–April	*Musim burung sengkaput* (season of Indian cuckoo)	–	Indian cuckoo (*Cuculus micropterus*) calls from the jungle	Considered to be a lean season for the Kedayan. Call of Indian cuckoo signifies the approaching of flowering season of local fruit trees in the following 2–3 months
April–May	*Musim taduh tahun* (Calm season of the year)	–	Calm waves in the sea	Calm waves in the sea signifies favorable time for fishing. There would be abundance of fish such as *bebatik* (*Gnathanodon speciosus*), *usus* (*Sillago sihama*) and *gulama* (*Johnius coitor*)
	*Musim bubuk* (Krill season)	–	–	Krill move closer inland where the waves are not strong. Harvesting krill is a major cultural and economic activity for the Kedayan. Notable food made from krill is *belacan* or shrimp paste
May–June	*Musim angin punay* (season of emerald dove wind)	–	A continuous wind with scattered drizzles coupled with the sighting of emerald dove (*Chalacophaps indica*)	The emerald doves are ‘skinny’ at this time of the year, and thus unfit for hunting. According to Maxwell [12], the wind could be alternatingly strong and calm *Angin punay* indicates imminent arrival of paddy season
June	*Musim bunga* (Flowering season)	–	–	A season influenced by the southwest monsoon. Local fruit trees begin flowering before the onset of mass fruiting season
June–July	*Payama padi/musim padi* (Paddy season)	Rising of Pleiades above the horizon in the dawn. Subsequently, rising of *rahang* is observed. Lastly, *binkasan* is observed	The blooming of *salimbudan* (*Mussaenda glabra* Vahl) indicates the appropriate time for felling of trees and preparation of paddy field [12]	Rising of Pleiades marks the onset of paddy season. Around 4 weeks’ time is allocated for burning the vegetation and debris in the field as rainfall is sparse at this time of the year. These constellations are all important for planning agricultural activities
	*Musim pohon sadaman, marakit dan makubung berbuah* (*Sadaman*, *makubung* and *marakit* fruiting season)	–	Fruiting of *sadaman* (*Macaranga trachyphylla*), *marakit* (*Macaranga conifer*) and *makubung* (*Macaranga gigantea*)	During this time of the year, fruits of the trees are ripe and falling. Emerald doves migrate to fruiting localities, which concurrently occurs with the cutting and clearing of vegetation. After the emerald doves have finished consuming the fruits, they gain weight. While working in the paddy field, the Kedayan also trap the *punay* (dove) for subsistence
	*Musim makan tahun Kedayan* (Kedayan annual feast)	–	–	*Makan tahun* is conducted to celebrate good harvest of paddy. The Kedayan annual feast is a pre-Islamic indigenous thanksgiving/harvest festival usually conducted after the end of the paddy season
July	*Musim kulat tahun* (Mushroom season)	–	Copious blooming of *Agaricus* sp.	A season that brings plenty of rain (a rainy season) due to the influence of Southwest monsoon. *Agaricus* sp. bloom copiously in the ecotones close to Kedayan paddy fields. Collection of these mushrooms is a major activity for the Kedayan
July–August	*Musim rebung buluh* (Bamboo shoots season)	Rising of Pleiades	Growth of bamboo shoots (*Bambusa* spp.)	The season of bamboo shoots is also known as *kartika dalam tanah* (Pleiades inside the ground/soil) as it is associated with the position of *kartika* (Pleiades) star. The rising of Pleiades indicates that it is time for the young bamboo shoots to grow. If the Pleiades is not yet seen above the horizon, the bamboo shoots are still inside the ground and when it is observed above the horizon and passes the zenith, the bamboo shoots are ready for harvest. Here, the rising of Pleaides is considered as analogous to the growth of bamboo shoots. The rising of Pleiades also indicates fruiting of *Nypa fruticans*. Kedayan used to tap the Nypa infructescence in the twentieth century for its sweet nectar, which was consumed as such or converted into *gula anau*, a sweetener [11]
August	*Musim menyamai banih padi* (Paddy sowing season)		Fruiting of *salimbudan* (*Mussaenda glabra*) indicates the time to sow paddy seeds	The season of paddy sowing usually takes place during the hot humid categories before the coming of first wet season Setting of *Mussaenda* fruits calls for the sowing of paddy which has to be completed by the time *Mussaenda* is denuded of all bracts and is in fruiting [12]
August–October	*Musim buah* (Fruiting season)		Horseflies approach *terap* (*Artocarpus odoratissimus*) fruits	Season usually starts with the fruiting of *terap* (*Artocarpus odoratissimus*), followed by *cempedak* (*Artocarpus integer*) and *kembayau* (*Canarium odontophyllum*), *mambangan* (*Mangifera pajang*), and *durian* (*Durio* spp*.*). The approaching of *terap* (*Artocarpus odoratissimus*) fruits by horseflies indicate beginning of season. Season usually ends with the fruiting of *tampoi* (*Baccaurea macrocarpa*) and *rambai* (*Baccaurea motleyana*)
November–December	*Musim penyu bertelur* (Sea turtle season)	Full moon indicates best timing for turtle egg harvest	–	The Northeast monsoon brings abundant rain. Sea turtles arrive to nest and lay their eggs especially when the nights are clear or during full moon. *Bulan penuh* (13th to 15th of lunar calendar) or full moon is considered as the best time to harvest sea turtle eggs (*Chelonioidea* spp) as the night is clear and bright
	*Musim hayarga* (season of strong winds)	–	Strong winds flowing inland from the sea accompanied by huge waves in the sea	A season influenced by the Northeast monsoon when strong winds blow from the sea to the inland. This period therefore refers to the time of the year when the waves of the sea were huge and strong. Thus, there is limited possibility to engage in fishing

We adapt the definition of biocultural indicators proposed by McKemey et al. [30] to define local seasonal indicators as ‘predictable, obvious, seasonal events in the landscape that are temporal landmarks of cultural and ecological significance.’ We prefer not to use the term ‘biocultural indicators’ as the term ‘biocultural’ when used as a descriptor in a form other than ‘biocultural diversity’ leads to confusion owing to precedence of its usage in biocultural anthropology [43]. We could not document the Kedayan use of the *picula* flower (*Luffa* sp.) to reckon daytime and undertake daily farming activities that was previously documented by Maxwell [12]. Maxwell also documented that the blooming of *salimbudan* (*Mussaenda glabra* Vahl) indicated the arrival of time for felling of trees and preparation of paddy field (*patandahan padi*); the setting of *Mussaenda* fruits called for the sowing of paddy, which has to be completed by the time *Mussaenda* is denuded of all bracts and is in fruiting [12]. When *angin punay* or ‘emerald dove wind’ accompanies the emerald dove (*Chalacophaps indica*) to Kedayan landscape, it is time to commence paddy planting. Unlike the previous two examples, we could document the knowledge on *angin punay* from our knowledge keepers, perhaps due to the wide-spread prevalence of trapping of the doves for meat.

When it comes to agriculture and other landscape management activities, the local seasonal indicators helped the Kedayan prepare in advance for an oncoming events such as clearing of land for paddy cultivation. However, the precise date of the activity was determined by the stellar calendar. The knowledge on stars, star clusters and constellations we documented from our knowledge keepers echoes that of Maxwell [12]. Heliacal rising of the *bintang kartika* (Pleaides), *bintang rahang* (the ‘jaw’ part of Taurus constellation) and *bintang binkasan* (consisting of three stars of the Orion’s belt) were all important for planning agricultural activities. The Kedayan scouted for the first ‘rising’ of the *bintang kartika* (Pleiades) on the east that approximately coincides with the month of May. This signals the exact date of commencement of paddy planting. The rising of Pleiades is also connected to the sprouting of bamboo shoots (Table 2), and fruiting of *Nypa fruticans* whose infructescence was tapped for its sweet nectar [11]. When rising of Pleiades is observed above the horizon, preparation of paddy field begins. When Pleiades had passed the horizon (approximately in June), works on cutting and slashing of vegetation on paddy fields should have been completed. The passing of Pleiades over the zenith indicates the beginning of a short dry spell known as *kemarau* or *panas kartika* (the Pleiades’ dry spell), which lasts from a few days to a week or two. Therefore, this advantageous period is used to leave the felled trees to dry before the field can be set on fire. The setting of fire to fields is immediately followed by short spells of rain. When *bintang rahang* passes the zenith before dawn, it signals the beginning of a brief dry spell or *panas rahang*, which can last for 7 days or more than that of *panas kartika*. By this time, a few fields would have been ready for planting. The last constellation that acts as a guide to the timing of paddy season is *bintang binkasan*. When they pass the zenith, it marks the beginning of another dry spell which would last for 3 days and by this time, paddy is planted. When *kartika*, *rahang* and *binkasan* have set down in west, all preparatory works on the paddy fields should have been completed.

#### Hybridization of Kedayan lunar calendar with Islamic calendar

Before Islam arrived in Borneo, the Kedayan used their indigenous lunisolar calendar along with the stellar calendar. Following the Islamization of Kedayan, the Kedayan lunar calendar was hybridized with the Islamic calendar. According to our knowledge keepers, the current Kedayan lunar months are known by the collective term *bulan melayu*, though the individual lunar month names follow the Islamic calendar. However, the names for waning and waxing of the moon, associated taboos and beliefs and local ecological knowledge have been retained by the Kedayan till date. The Kedayan recognize various moon shapes such as *bulan baru* (new moon), *bulan luak* (crescent moon), *bulan separuh kalam* (half-moon) and *bulan purnama/bulan penuh* (full moon). The hybridization of calendars made it easier to calculate lunar months which otherwise would have required constant observation of moon phases. Also, the community today does not have to undertake complex intercalation required to synchronize solar and lunar calendars. Barring *makan tahun* and *makan arwah*, all Kedayan indigenous festivals have been replaced by Islamic festivals. *Makan tahun* is the thanks giving festival determined by the stellar calendar, while *makan arwah* is the feast to mourn the dead now held during the month of *syaban* of the Islamic calendar.

The first week of the *bulan melayu,* from 1st to 7th of Islamic lunar month is called as *pabani hidup*, and 8th to 22nd as *pabani habis*. The 23rd to 28th or 29th of the Islamic lunar calendar when the moon appears in as crescent is known as *pabani mati. Pabani* is a taboo period for farmers as they believe the insect pests will destroy crops if they venture to the paddy field during *pabani hidup* and *pabani mati.* Hence, it is best for them to go to the paddy field when *pabani* ends (*pabani habis, habis* = finished) during 8–22 of *bulan melayu*. However, there appears to be diverging understanding of this taboo. A Kedayan knowledge keeper reported that taboo for farmers only falls during *pabani mati*, at the last week of the month (i.e., 23rd to 28th or 29th); insisting to work on the paddy fields might lead to crop damage by wild animals, birds or insects. Another knowledge keeper reported that the term *pabani* is also associated with the spring and neap tide. The spring tide occurs during new moon until full moon and the neap tide occurs 7 days after spring tide, which is on the 23rd of lunar calendar. Spring tide of the sea is the most preferable period for the farmers to do planting activities as compared to neap tide. The Kedayan believe that planting activities conducted during neap tide would only end up in crop lodging and bad harvest.

The Kedayan also associate the Islamic lunar calendar with harvesting of non-agricultural resources such as crabs (*Scylla serrata*) and *umbut*, the cabbage of coconut palm (*Cocos nucifera* L.)*.* Crabs (mostly egg bearing females) are meaty when the new moon starts to grow until it almost reaches full moon (1st to 12th). During full moon (13th to 15th), there will be a mixture of meaty males and females, and toward the end of lunar month (23rd to 28th or 29th) most harvested crabs would be males and less meaty. The Kedayan harvest *umbut* (coconut palm cabbage) from the new moon until the moon reaches its full rounded shape (1st to 15th). Apart from crab and palm harvesting season, the Kedayan also specify that it is best to hunt animals such as *pelanduk* (mouse deer, *Tragulus javanicus*)*, kijang* (muntjac deer, *Muntiacus atherodes*) and *payau* (barking deer, *Muntiacus muntjak*) during *bulan luak* and *bulan separuh kalam* (1st to 12th and 17th to 29th) when the moon is in crescent shape. Ramlee’s study of 2006 also corroborates this [9]. *Bulan penuh/bulan purnama* or full moon is to be avoided as the conditions are too bright, making it difficult to catch animals. *Bulan penuh* (13th to 15th) or full moon, however, is considered as the best season to harvest sea turtle eggs (*Chelonioidea* spp*.*) as the night is clear and bright. This window of egg collection, however, is only available for a short period in a year.

### Contemporary relevance and popularity of Kedayan ecological calendar

Questions 7–10, and 14–17 in the questionnaire (Additional file 1) were designed to study to test the relevance and popularity of Kedayan ecological calendar today. The findings reveal that very few respondents (*n* = 27, 25.3%) are aware of the existence of Kedayan LEC: 80 respondents (74.7%) reported of not being aware of its existence. A very small amount number of respondents reported to be fully knowledgeable of the calendar (*n* = 4, 3.7%). Majority (*n* = 63, 58.9%) reported that the calendar is not important (Additional file 2). Others (*n* = 44, 41.1%) stated that Kedayan LEC is important despite of having weak knowledge related to it. Thirty-one respondents (29%) claimed that it is important to conserve the Kedayan local knowledge related to the calendar, while one of them stated that the calendar indicated the best time to pursue activities such as foraging and hunting. Negligible number of respondents found it useful to determine Islamic events (*n* = 1), to prevent disasters (*n* = 1), and as novelty for the Kedayan and other communities (*n* = 1). One respondent stated that to his knowledge, there are only two calendars (Gregorian and Islamic); two respondents claimed that modernization have caused the calendrical practices to be unviable and another respondent asserted that poor transmission of calendric knowledge has led to loss of its significance. Forty-five respondents (42%) did not provide any reasons why the Kedayan ecological calendar is not important. We found no relationship between education levels of our respondents, their occupation and perception of importance of Kedayan calendar (Tables 3, 4).

**Table 3:** Pairwise correlations for education (Q5) and perceived importance of Kedayan calendar (Q14)

Variables	Importance of Kedayan calendar (Q14)
Primary school	− 0.115 (0.237)
Secondary school	0.112 (0.250)
Diploma	− 0.067 (0.490)
Undergraduate	− 0.040 (0.686)
Postgraduate	− 0.007 (0.940)
Ph.D.	0.116 (0.233)
Other	− 0.027 (0.783)

There is no statically significant correlation between education and people’s perception of importance of Kedayan calendar

***p < 0.01, **p < 0.05, *p < 0.1

**Table 4:** Pairwise correlations between occupation (Q6) and perceived importance of Kedayan calendar (Q14)

Variables	Importance of Kedayan calendar (Q14)
Government sector	− 0.032 (0.741)
Private sector	− 0.109 (0.265)
Fishing	− 0.144 (0.138)
Other	0.209** (0.031)

There is a very weak and statistically significant correlation between people’s perception of importance of Kedayan calendar and other occupations (*r* = 0.209, *p* < 0.05) alone. These results indicate that the occupations are not correlated with how the people perceive the importance of Kedayan calendar (Q14)

***p < 0.01, **p < 0.05, *p < 0.1

#### Calendric knowledge of the contemporary Kedayan

Given that the total respondents recruited in this study was 107, the number of correct responses for Kedayan months was relatively low (Table 5): *Musim rebung* or season of bamboo shoot (*n* = 33, 31%), *hayarga* (*n* = 22, 21%), *angin punay* or emerald dove wind (*n* = 21, 20%) and *payama padi* (*n* = 18, 17%). There is a weak correlation between respondent’s self-professed awareness of Kedayan calendar, and actual knowledge of the calendar (*r* = 0.339, *p* < 0.05) (Table 6). For respondent’s ability to name the correct Kedayan month, we found a very weak and statistically significant correlation between self-professed awareness of Kedayan calendar and people recognizing only *payama padi* as Kedayan month/season (*r* = 0.199, *p* < 0.05). However, the overall results indicate that people who expressed self-proficiency in the Kedayan calendar (Q7 and Q8) are not fully aware of Kedayan months/seasons.

**Table 5:** Respondents’ ability to name Kedayan months/seasons

Name of Kedayan month	No. of responses (%)
*Musim rebung* (season of bamboo shoot)	33 out of 107 (31%)
*Hayarga*	22 out of 107 (21%)
*Angin burung punay* (Wind of Emerald dove)	21 out of 107 (20%)
*Payama padi* (paddy season)	18 out of 107 (17%)

**Table 6:** Pairwise correlations between Q7, Q8, and Q10

Variables	Awareness of Kedayan calendar (Q7)	Knowledge of Kedayan calendar (Q8)
Knowledge of Kedayan calendar (Q8)	0.339** (0.000)	
*Payama padi* (Q10 answer No 3)	0.199** (0.040)	0.175 (0.072)
*Angin burung punay* (Q10 answer No 8)	− 0.016 (0.868)	0.151 (0.121)
*Hayarga* (Q10 answer No 7)	0.184 (0.058)	0.144 (0.140)
*Musim rebung* (Q10 answer No 9)	− 0.108 (0.266)	0.082 (0.403)

There is a weak and statistically significant correlation between self-professed awareness of Kedayan calendar and knowledge of Kedayan calendar (*r* = 0.339, *p* < 0.05). There is also a very weak and statistically significant correlation between awareness of Kedayan calendar and people recognizing only *payama padi* as Kedayan month/season (*r* = 0.199, *p* < 0.05). The overall results indicate that people answered who expressed self-proficiency in the Kedayan calendar (Q7 and Q8) are not fully aware of Kedayan month/season

***p < 0.01, **p < 0.05, *p < 0.1

*Musim rebung* (season of bamboo shoots) received the highest number of responses. This could be because *rebung* or bamboo shoot is still being harvested by the Kedayan up to present day. *Hayarga* has the second highest number of responses. For the Kedayan, this month is often associated with the season of strong winds blowing from the ocean to inland signifying unfavorable time for fishing activities. *Angin punay* has the third highest number of responses, after *hayarga*. The respondents who chose *angin punay* associated their knowledge to the emerald dove (*burung punay*) that are snared for their meat. With the complementary information from the qualitative interviews, the presence of *burung punay* could also be linked with the fruiting and ripening of *sadaman* (*Macaranga trachyphylla*)*, makubung* (*Macaranga gigantea*) and *marakit* (*Macaranga conifera*) trees, as the birds feed on their fruits. The arrival of *angin punay* also signifies that the Kedayan paddy season is about to begin. From this, it is understandable that knowledge and dependency on local seasonal indicators (calendric plants and animals), and familiarity with the Kedayan seasons is still prevalent in a small section of the community. The lowest number of responses of the Kedayan month was on *payama padi* or paddy season. Although there were respondents who chose *payama padi* as the Kedayan month or season, those respondents were not engaged with agriculture. Thus, it can be inferred that some of the respondents still possess the calendric knowledge on *payama padi* although they no longer are involved in agriculture. The low number of responses on *payama padi* could also be attributed to many Kedayan farmers having abandoned agriculture (paddy farming) and have started working in government (*n* = 36 respondents) and private sectors (*n* = 28 respondents).

To gauge respondents’ adherence to Kedayan traditions and knowledge on calendar and calendric practices indirectly, we used their knowledge on appropriate timings to consult healers as proxy (questions 15 & 16). Findings reveal only 35% (*n* = 37; *m* = 19, *f* = 18) of the respondents have consulted healers before, while 65% (*n* = 70; *m* = 25, *f* = 45) of them reported to have never consulted a healer before.

We found no statistically significant correlation between consulting healers, knowledge on time requisite to consult healers, and self-professed awareness and knowledge of Kedayan calendar (Table 7). Twenty-four (22%) respondents stated that there is auspicious time to consult healers while a majority 83 (78%) opined otherwise. The 24 respondents cited Friday night and post-Isya’ (night prayer for the Muslims) (*n* = 3), evenings (*n* = 2), Friday and Saturday nights (*n* = 1), any day except Wednesday (*n* = 1), Tuesday (*n* = 1), Thursday (*n* = 1), Friday (*n* = 1) and Saturday night (*n* = 1) as favorable time to consult the healer. Two respondents could not name specific time, although they acknowledged the existence of appropriate timings. The two respondents who named evenings as appropriate time to consult healers linked it to higher chances for availability of the healer at home.

**Table 7:** Pairwise correlations between consulting the healer (Q15), the time (Q16), awareness (Q7) and self-professed knowledge on calendar (Q8)

Variables	Awareness of Kedayan calendar (Q7)	Knowledge of Kedayan calendar (Q8)
Consult healer (Q15)	− 0.060 (0.536)	− 0.040 (0.685)
Time to consult healer (Q16)	0.100 (0.304)	− 0.106 (0.277)

This table shows that there is no statistically significant correlation between consulting healers, knowledge on time requisite to consult healers, and self-professed awareness and knowledge of Kedayan calendar

***p < 0.01, **p < 0.05, *p < 0.1

#### Demographic particulars influencing calendric knowledge

Our study finds that level of formal education does not determine self-professed awareness and knowledge on Kedayan calendar (Table 8). Likewise, there is no correlation between respondents’ occupations, and self-professed awareness and knowledge of Kedayan calendar (Table 9).

**Table 8:** Pairwise correlations between education levels (Q5) and Q7, Q8

Variables	Self-professed awareness of Kedayan calendar (Q7)	Self-professed knowledge of Kedayan calendar (Q8)
Primary school	0.079 (0.420)	− 0.027 (0.781)
Secondary school	0.083 (0.396)	0.236** (0.014)
Diploma	− 0.147 (0.131)	− 0.103 (0.291)
Undergraduate	− 0.003 (0.976)	− 0.106 (0.277)
Postgraduate	− 0.039 (0.692)	− 0.063 (0.517)
Ph.D.	0.167 (0.085)	− 0.019 (0.845)
Other	0.032 (0.746)	− 0.033 (0.732)

There is a very weak and statistically significant correlation between secondary school education and self-professed knowledge of Kedayan calendar (*r* = 0.236, *p* < 0.05) alone. The overall results indicate that level of formal education does not determine self-professed awareness and knowledge on Kedayan calendar

***p < 0.01, **p < 0.05, *p < 0.1

**Table 9:** Pairwise correlations between occupation (Q6) and Q7, Q8

Variables	Self-professed awareness of Kedayan calendar (Q7)	Self-professed knowledge of Kedayan calendar (Q8)
Government sector	0.133 (0.173)	0.172 (0.076)
Private sector	− 0.101 (0.300)	− 0.005 (0.957)
Fishing	− 0.067 (0.495)	− 0.052 (0.594)
Other	− 0.004 (0.969)	− 0.140 (0.149)

***p < 0.01, **p < 0.05, *p < 0.1

The age group of 50 to 59 years old had the highest number of respondents who chose the right name for Kedayan months: *musim rebung* (*n* = 12 responses), *hayarga* (*n* = 12), *angin punay* (*n* = 8) and *payama padi* (*n* = 8). Interestingly, some younger respondents from the age group of 19–29 years old also answered the correct Kedayan months: *musim rebung* (*n* = 8 responses), *hayarga* (*n* = 2), *angin burung punay* (*n* = 5) and *payama padi* (*n* = 3). Majority of the respondents who had provided correct answers for names of Kedayan months had received formal education at least to the secondary level. *Musim rebung*: secondary education (*n* = 18), degree holders (*n* = 5), diploma (*n* = 4) and primary (*n* = 2), master (*n* = 2) and other (*n* = 2). *Hayarga:* secondary (*n* = 12), diploma (*n* = 3), masters (*n* = 2) and primary (*n* = 1), degree (*n* = 1), PhD (*n* = 1) and other educational background (*n* = 1). *Angin punay:* secondary (*n* = 13), diploma holders (*n* = 4), undergraduate (*n* = 2) and masters (*n* = 2). *Payama padi:* secondary (*n* = 10), diploma (*n* = 2) and master (*n* = 2), primary (*n* = 1), undergraduate (*n* = 1), PhD (*n* = 1) and others (*n* = 1). Occupation-wise, most of the respondents who gave right names of Kedayan months were employed in the Government sectors: *musim rebung* (*n* = 15), *hayarga* (*n* = 14), *angin punay* (*n* = 12), *payama padi* (*n* = 12). Only few of the successful responses were from those engaged in fishing (*musim rebung n* = 3 responses, and *payama padi n* = 1).

#### Transmission of calendric knowledge

**Fig. 4:**
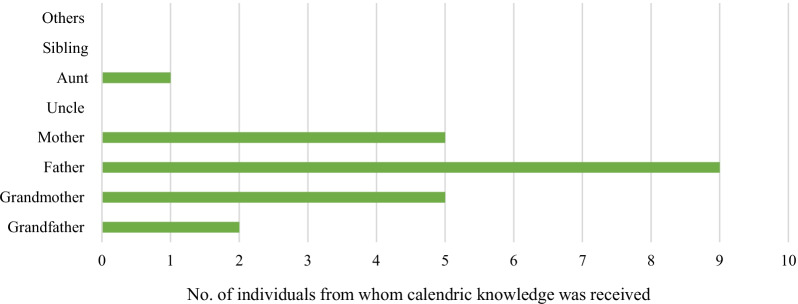
Community members from whom calendric information was received. *Note*: The chart shows that no members had received knowledge from a knowledge keeper outside their family

Our study shows that only 14 (13.1%) of the respondents had received some form of calendric knowledge (Fig. 4). A vast majority (86.9%; *n* = 93) reported to have never received any. For the 14 respondents who had received some form of calendric knowledge, transmission of calendric knowledge from the father is the highest (9 responses), followed by grandmother and mother (5 responses). Two respondents stated that they received calendric knowledge from grandfather and one from aunt. Only 2 respondents (1.9%) had transmitted some form of calendric knowledge to other people. Thus, existing people-to-people transmission of calendric knowledge is family centric, and limited to receiving alone with just two people reporting to have transmitted some calendric knowledge.

## Discussion

### The Kedayan calendar linked skyscape and biodiversity with sociocultural aspirations to foster adaptive management of landscape

**Fig. 5:**
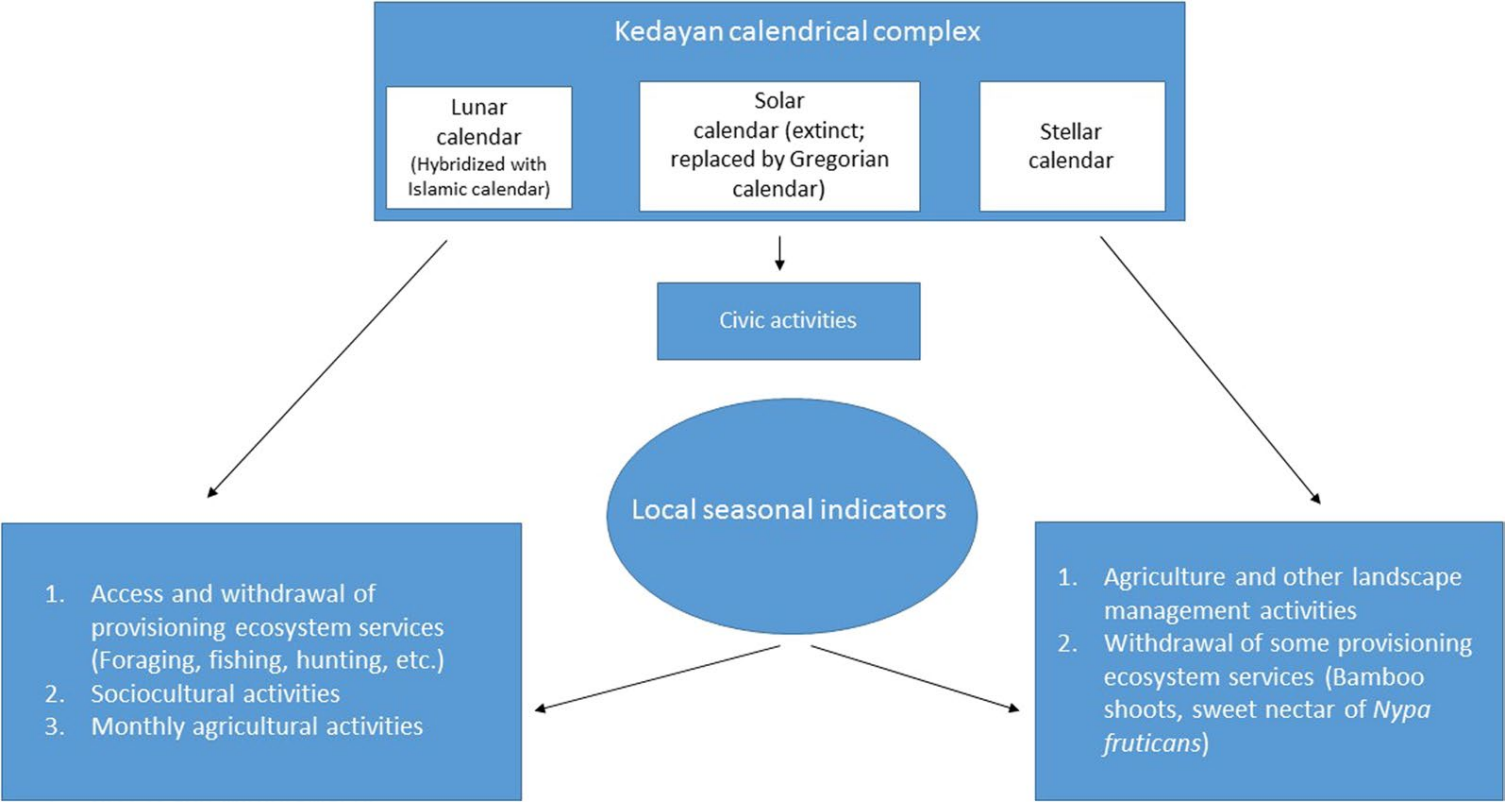
Schematic representation of activities facilitated by the Kedayan ecological calendar

In the past, the Kedayan LEC linked the landscape, and its flora and fauna to the social and cultural rhythms of the community, forming an important component of biocultural diversity [23] (Fig. 5). This ability of the Kedayan LEC to culturally link temporal and spatial scales could help the community even today in adaptive management of their landscape [14–17]. The Kedayan were traditionally agriculturists or paddy growers who also partake in seasonal hunting and fishing activities [11, 40]. Agriculture is a major land use all over the world that influences land cover and vegetation pattern [44–46]. Many subsistence farming communities have relied on their LEC to manage their landscape through agriculture [47–50]. To be successful in agriculture, the Kedayan relied on the knowledge of seasons, weather patterns, and phenology of plants and animals disbursed by the ecological calendar. LECs are also frameworks for accessing and withdrawing ecosystem services [19], a dimension that is often overlooked. The (lunar) calendar informs the Kedayan about the appropriate time to harvest coconut palm cabbage, crabs and also hunt wild animals, facilitating withdrawal of provisioning ecosystem services. Lunar phases influence animal and plant rhythms [51–53], the knowledge of which disbursed through calendars help the communities harvest resources efficiently. In the rainy nights of 1970s, the Kedayan hunted mouse deer that took refuge in the drylands offered by hillocks [2]. For a successful deer jacking, the Kedayan relied on their calendar to choose dark nights. In addition, the mobilizing of a hunting party would have also been easier when the rain is forecasted through the calendar. The Kedayan LEC triangulated the position of sun, moon and stars with local seasonal indicators such as loofah (*Luffa* sp.), emerald dove and *Mussaenda* sp. to disburse precious local knowledge that helped the Kedayan engage in agriculture, foraging, hunting and fishing successfully [12], and also plan sociocultural events such as festivals and rituals.

Throughout the world, local communities have used heliacal apparitions as temporal landmarks determine the onset of seasons [13, 54]. Pleiades and Orion especially are of paramount significance in many LECs, helping communities to forecast monsoon patterns with high precision and adapt agricultural activities accordingly [13, 19, 28, 54]. Communities such as the Kanekes (Baduy) of Indonesia [47], and the Mao Naga of Northeast India [39] have dedicated calendar keepers who collate and process the observatory data from skyscape and landscape, and disburse it to the community to facilitate sociocultural activities. Although the Kedayan do not have dedicated calendar keepers, the Kedayan healers and elders knowledgeable in the ecological calendar serve as de facto calendar keepers. For the Kedayan, the rising of Pleiades and the sprouting of bamboo shoots are connected events happening in the same landscape, as celestial events are capable of influencing the rhythms of plants and animals. This is understood from the term *kartika dalam tanah* meaning ‘Pleiades under the soil’ that lexicalises season of bamboo shoots [21]. Indeed, local communities conceptualize and experience skyscape, landscape and climatological phenomena as interlinked processes [13, 55, 56]. Communities such as the D(L)akota of Minnesota and Kodi of Indonesia see changes in the landscape as mirror images of those of the skyscape [57, 58]. For the D(L)akota, the behavior of fisher (*Pekania pennanti*) mirrors the circumpolar motion of the Big Dipper star cluster of Ursa Major [59]. Such conceptualization contrasts formal academic approaches of considering skyscape and landscapes separately.

Phenology of local seasonal indicators such as flora and fauna is influenced by temperature, diurnal variation, etc., prevailing in the landscape, and any disturbance to the landscape [17, 60]. Thus, local seasonal indicators are dynamic, capable of reflecting the changing conditions of the landscape, a property that makes them useful in adaptive management of landscapes [14, 17, 34]. Generally, the incorporation of local seasonal indicators offers much adaptability to LECs which would have otherwise been rigid, by virtue of being tied to the regular astronomical events [61]. For the Kedayan however, observation of local seasonal indicators provided advance information on the need for watching the skyscape closely for heliacal apparitions. Maxwell [12] records that in the 1970s, the Kedayan used to ready themselves for swidden agriculture when they observed local seasonal indicators (e.g., *angin punay*). However, the activities officially commence in full rigor in a synchronized manner only when the rising of *kartika* (Pleiades) is observed. This also ensured that the crop in all fields matured simultaneously; fields with crops maturing late face the onslaught of insect pests whose population would have peaked already [12]. As the Kedayan live in a landscape forested with secondary forests, spotting constellations and star clusters such as the Pleiades is not easy. And, Kedayan elders constantly sent messengers toward open areas from where the star cluster can be spotted. Thus, the advance information from local seasonal indicators made it easier for scouting for heliacal apparitions which was quintessential to carry out agriculture.

### Breakdown of knowledge transmission explains loss of local ecological calendar

Our study shows that the Kedayan LEC has lost the lunisolar calendric components. Remnant knowledge on star clusters, constellations and local seasonal indicators is limited to a few knowledge keepers. Majority of the community members are not even aware of the existence of the Kedayan ecological calendar and even opined that the calendar is not important anymore. The political patronage and popularity enjoyed by the Gregorian and Islamic calendars and their popularity among the community may explain the lack of awareness on Kedayan ecological calendar. Calendric knowledge like other forms of local knowledge is dynamic, changing with the progression of time and broadening of community networks [62]. However, total replacement of LEC by politically powerful standardized calendars is of great concern, as losing a LEC is tantamount to losing a repository of knowledge [14]. For instance, in rural areas of Pakistani Punjab, Gregorian calendar is the civil calendar while Islamic calendar determines religious activities and festivals. Together, they have contributed to the loss of Pakistan’s local ecological calendars [63]. Also, LECs all over the world are losing their cultural importance as people leave traditional occupations such as subsistence farming that warrant fine knowledge of local weather patterns.

In the Iban community of Brunei Darussalam, traditional occupations such as subsistence farming and hunting are positively correlated with retention of local knowledge [64]. Our results do not show any positive association between Kedayan engaged in agriculture and calendric knowledge. The Kedayan calendar suffers from breakdown of transmission of calendric knowledge from knowledge keepers to the general community members. Even the negligible transmission existing today is confined to the family spheres, with people hardly transmitting knowledge to others. Calendric knowledge is an elite form of specialist knowledge that when combined with rituals helps the calendar keepers wield considerable power in the society [39, 65–69]. Calendar keeping in classic societies such as the Maya and the Nahua was a closely guarded secret, governed by strict codes of practice typical of codified knowledges [65]. The Mesopotamian calendars had specific manuals for the calendar keepers engaged in moon watching [70]. In communities with dedicated calendar keepers, calendars were practiced, conserved and perpetuated by the calendar keepers, and revitalizing the institution of calendar keeping could revitalize the calendars [39]. Unlike such codified calendars, in communities with no dedicated calendar keepers, calendric knowledge prevails in the collective memory of the community, even when there are certain specialists knowledgeable about calendric practices. Thus, in such societies, revitalizing the calendar would require revitalizing transmission pathways of knowledge. Collective memory reinforced through folklores [71–73] plays an important role in sustaining folk calendars. Folklores are also mediums of transmission of calendric knowledge. Examples include the Great Oak calendric myth of the Baltic Finns conveying the annual solar and vegetation cycle [74], calendric rituals conveyed through the folk songs of Kriashens of Mamadysh [75] and the Eastern Bengal ballads (*baramashi*) transmitting calendric knowledge [76]. Decoding the knowledge embedded in calendric folklores can foster reconstruction and revitalization of calendars. That the Kedayan once had *pantun* (poems) communicating calendric knowledge is understandable from Maxwell [12]. However, we recorded no calendric stories, songs, riddles or puzzles related to the Kedayan calendar, indicating that they have been lost. The Kedayan of neighboring Malaysian Borneo region appear to have retained many indigenous calendric practices and knowledge [77]. Collaborating with the Kedayan of Malaysian Borneo could unearth calendric folklores and knowledge that the Kedayan of Brunei have lost.

The Kedayan healers are relatively knowledgeable in the calendar than other members of the society owing to the intersection of calendric knowledge and healing knowledge/practices [40]. In communities with no dedicated calendar keepers, there has to be active transmission of knowledge from the knowledgeable elders such as the healers to the general public, and vice versa. In addition, collective calendar keeping requires constant flow of observatory knowledge on local seasonal indicators from the community members to the knowledge keepers [39]. For the calendar to be alive in the collective memory, calendric knowledge has to be frequently retrieved, discussed and transmitted [58, 78]. Our study shows that there is a total breakdown of transmission in the Kedayan community. The negligible intra-family transmission is inadequate for sustaining calendric knowledge and practices. The breakdown of community transmission of calendric knowledge explains the fading popularity of the Kedayan local ecological calendar.

## Conclusions

The dynamic local ecological calendar of Kedayan documented through this study is an embodiment of Kedayan local knowledge. The calendar is dynamic, changing with the environmental and social conditions of the Kedayan communities. It is also highly contextualized and localized to the study area. The Kedayan calendar significantly represents plants and animal behaviors, the availability of seasonal resources and sociocultural events. Local seasonal indicators such as bird calls, appearance of birds, nature of sea, etc., provide cues on the onset of seasons. Indicators also cue on the time to scout for star clusters that act as temporal markers for commencement of agricultural activities. The calendar provides information on the availability of resources such as mushrooms, favorable time for fishing and hunting, etc., thereby also acting as a framework for access and withdrawal of provisioning ecosystem services. By virtue of being relevant to the local ecology and culture, LECs such as the Kedayan calendar differ from the standardized calendars that do not have the mandate to be relevant to the local ecological conditions and sociocultural requirements of local communities. However, our study finds that despite its ecological and sociocultural relevance, much of the calendric knowledge has been lost. Very few respondents (*n* = 27, 25.3%) are aware of the existence of Kedayan LEC, with majority (*n* = 80, 74.7%) not being aware of its existence. Majority of respondents (58.9%) also reported that the calendar is not important. Respondents who expressed self-proficiency in the Kedayan calendar are not fully aware of Kedayan months/seasons, indicating an inflated sense of self-proficiency in the calendar. Our study fails to find any significant correlation between people who have consulted folk healers before, knowledge on culturally appropriate time requisite to consult healers, and self-professed awareness and knowledge of Kedayan calendar. This shows that even people who are positively inclined toward traditions such as consulting a folk healer are not proficient in the LEC. Only 14 (13.1%) of the respondents interviewed by us had received some form of calendric knowledge, and a negligible 2 (1.9%) alone reported to have transmitted calendric knowledge. This indicates a total breakdown in transmission of calendric knowledge among the Kedayan community, which in turn explains the fading popularity of the Kedayan local ecological calendar. The Kedayan local ecological calendar is an ecocultural heritage of the community that when revitalized through community initiatives could foster adaptive landscape management, and access and withdrawal of provisioning ecosystem services. The community will have to invest in enhancing transmission of calendric knowledge for any revitalization efforts to be successful.

## Supplementary Information

**Additional file 1.** Questionnaire for determining the relevance and popularity of the Kedayan ecological calendar, and its transmission in the community.

**Additional file 2.** Summary of results of the interviews using structured questionnaire.

## Data Availability

Data supporting reported results are included in the article and the Additional file 2.

## Abbreviations

Sp(p): Species
LEC: Local ecological calendars
