# Supplementary material for: The fading popularity of a local ecological calendar from Brunei Darussalam, Borneo
Source: J Ethnobiol Ethnomed. 2022 Apr 16;18:33. doi: 10.1186/s13002-022-00525-9 (PMC9013451; doi:10.1186/s13002-022-00525-9)
Supplement: Supplementary file 1 — Additional file 1. Questionnaire for determining the relevance and popularity of the Kedayan ecological calendar, and its transmission in the community. [file 13002_2022_525_MOESM1_ESM.docx]

**Additional file 1. Questionnaire for determining the relevance and popularity of the Kedayan ecological calendar, and its transmission in the community**

Section A: Personal particulars

1. Age

🞎 18 below

🞎 19-29

🞎 30-39

🞎 40-49

🞎 50-59

🞎 60- 69

🞎 70 above

gender :

2. Are you Kedayan?

🞎 Yes

🞎 No

3. If YES, please choose options below

🞎 Your father is Kedayan and your mother is not Kedayan

🞎 Your mother is Kedayan and your father is not Kedayan

🞎 Both of yours parents are Kedayan

🞎 Others_______________(specify)

4. Which locality you belong to? State the name of village and district

🞎 Brunei Darussalam (Village _______________District _______________)

🞎 Sarawak (Village______________District_______________)

🞎 Sabah (Village_______________District_______________)

🞎 Labuan (Village_______________District_______________)

🞎 Others (Village_______________District_______________)

5. Educational background

🞎 Primary school

🞎 Secondary school

🞎 Diploma

🞎 Undergraduate

🞎 Postgraduate

🞎 PhD

🞎 other_______________ (specify)

6. Occupational background

🞎 Government sector

🞎 Private sector

🞎 Business

🞎 Agriculture (specify what kind of crop you cultivate: (………………………………………..)

🞎 Hunting (specify what kind of animals you hunt: (…………………………………………….)

🞎 Fishing

🞎 Herbalist (healer)

🞎 Other (specify)……………….

Section B: Knowledge on Kedayan traditional calendar

7. Are you aware of the existence of Kedayan traditional calendar?

🞎 Yes

🞎 No

8. Are you fully knowledgeable about the Kedayan calendar?

🞎 Yes

🞎 No

9. Please tick ✓ the name of Kedayan months according to your knowledge.

| January |  |
| --- | --- |
| Muharram |  |
| Safar |  |
| March |  |
| June |  |
| Jamaddil Awal |  |
| November |  |
| Sya’ban |  |
| December |  |
| Ramadan |  |
| April |  |
| Syawal |  |
| Don’t know |  |

10. Please tick ✓the names of Kedayan seasons according to your knowledge.

| Pabani hidup |  |
| --- | --- |
| Panas |  |
| Payama |  |
| Tengkujuh |  |
| Nawang |  |
| Pabani mati |  |
| Hayarga |  |
| Musim angin punay |  |
| Musim rebung |  |
| Don’t know |  |

12. Have you ever-received information related to Kedayan calendar from anybody (including Kedayan days, months and seasons)?

🞎 Yes

🞎 No

If you pick YES, from whom you had received the information?

🞎 Grandfather

🞎 Grandmother

🞎 Father

🞎 Mother

🞎 Uncle

🞎 Aunt

🞎 Siblings

🞎 Others (please specify)……….

13. Have you ever-transmitted knowledge on Kedayan traditional calendar to anybody?

🞎 Yes

🞎 No

If you pick YES, to whom which you had transmitted the Kedayan traditional calendar knowledge?

🞎 Grandfather

🞎 Grandmother

🞎 Father

🞎 Mother

🞎 Uncle

🞎 Aunt

🞎 Siblings

🞎 Children

🞎 Others (please specify)……….

14. Do you think Kedayan calendar is important?

🞎 Yes

🞎 No

If you pick YES, please specify why? …………………………………………………………………………………………….

If you pick NO,

Have you face any difficulties due to your lack of knowledge on Kedayan calendar?

🞎 Yes

🞎 No

Please specify………………………………………………………………………………………

15. Have you ever consulted a Kedayan traditional healer in the past?

🞎 Yes

🞎 No

If yes, Please specify how many times ………………………………………….

16. Do you think there is a best time/ day to consult the healer? (e.g. Mondays, Fridays, etc.; Morning/ evening etc; full moon night, Crescent night, etc).

🞎 Yes

🞎 No

If yes, Please specify………….

17. If you have answered yes to the above question, please specify why the time/day you mentioned is the most appropriate.

🞎 Convenience (holiday/ off time)

🞎 other reasons (s) _____________________________
