# Supplementary material for: The fading popularity of a local ecological calendar from Brunei Darussalam, Borneo
Source: J Ethnobiol Ethnomed. 2022 Apr 16;18:33. doi: 10.1186/s13002-022-00525-9 (PMC9013451; doi:10.1186/s13002-022-00525-9)
Supplement: Supplementary file 2 — Additional file 2. Summary of results of the interviews using structured questionnaire. [file 13002_2022_525_MOESM2_ESM.docx]

**Additional file 2.** Summary of results of the structured interviews.

|  | Characteristics | Responses | No. of respondents | Percentage |
| --- | --- | --- | --- | --- |
|  | Aware on the existence of Kedayan ecological calendar? | Yes  No | 27  80 | 25.3%  74.7% |
|  | Received knowledge related Kedayan ecological calendar?  If yes, from whom? (Some respondents picked more than one answer) | Yes  No  Grandfather  Grandmother  Father  Mother  Uncle  Aunt  Siblings  Others | 14  93  2  5  9  5  0  1  0  0 | 13.1%  86.9%  2%  5%  8%  5%  0%  1%  0%  0% |
|  | Are you fully knowledgeable of Kedayan ecological calendar? | Yes  No | 4  103 | 3.7%  96.3% |
|  | Ever transmitted knowledge related to Kedayan ecological calendar to anybody?  If yes, to whom? | Yes  No  Grandfather  Grandmother  Father  Mother  Uncle  Aunt  Siblings  Children  Others | 2  105  0  0  0  0  0  0  0  1  1 | 1.9%  98.1%  0%  0%  0%  0%  0%  0%  0%  1%  1% |
|  | Is Kedayan calendar important?  If yes, why?  If no, why? | Yes  No  Conserve the local knowledge of Kedayan  Indicate best time to pursue cultural activities (eg. Full moon; harvesting sea turtle eggs, harvesting edible palm and catching crabs as these resources are considered ‘big’ and ‘round’)  To determine Islamic events  Prevent ‘disaster’  New knowledge for the Kedayan and communities in Brunei Darussalam  Not available  First time to hear about Kedayan ecological calendar  There are only two (2) calendars (Gregorian and Islamic)  Modernization have caused Kedayan ecological calendar to be impracticable  Poor transmission of Knowledge  Not available | 44  63  31  1  1  1  1  9  14  1  2  1  45 | 41.1%  58.9%  29%  1%  1%  1%  1%  8%  13%  1%  2%  1%  42% |
|  | Have you ever met a Kedayan healer before?  If yes, how many times?  Is there a specific time to meet healer?  If yes, when? | Yes  No  Once  Twice  More than twice  When necessary  Often  Not available  Yes  No  Evening  Best time and day  Friday night and after Isya’  Friday night and Saturday night  Everyday except Wednesday  Tuesday, Thursday and Friday  Every Saturday night  Not available | 37  70  4  2  18  1  4  8  24  83  2  2  3  1  1  1  1  13 | 35%  65%  4%  2%  17%  1%  4%  7%  22%  78%  2%  2%  3%  1%  1%  1%  1%  13% |
